# E6/E7 Similarity to Human Papillomavirus Prototypes and Performance of HPV Testing by Cobas 4800 HPV Test and Anyplex II HPV HR

**DOI:** 10.1002/jmv.70668

**Published:** 2025-10-28

**Authors:** Luani R. Godoy, Mariam El‐Zein, Piet Cools, Elizaveta Padalko, Bo Verberckmoes, Olivier Degomme, Heleen Vermandere, Laila Sara Arroyo Mühr, Milan S. Stosic, Eduardo L. Franco, Adhemar Longatto‐Filho, the ELEVATE study group, Vivian Alejandra Neira, Sónia Dias, Ana Gama, Bernardo Jose Vega Crespo, Yasmin M. Guimarães, Tauana C. Dias, Rui Manuel Reis

**Affiliations:** ^1^ Division of Cancer Epidemiology McGill University Montréal Quebec Canada; ^2^ Molecular Oncology Research Center Barretos Cancer Hospital Barretos São Paulo Brazil; ^3^ Department of Diagnostic Sciences, Faculty of Medicine and Health Sciences Ghent University Ghent Belgium; ^4^ Department of Clinical Biology, Microbiology and Immunology Ghent University Ghent Belgium; ^5^ International Centre for Reproductive Health, Department of Public Health and Primary Care, Faculty of Medicine and Health Sciences Ghent University Ghent Belgium; ^6^ Center for Cervical Cancer Elimination F56, Karolinska University Hospital, Karolinska Institutet Huddinge Sweden; ^7^ Department of Life Sciences and Health, Faculty of Health Sciences Oslo Metropolitan University Oslo Norway; ^8^ Department of Microbiology and Infection Control Akershus University Hospital Lørenskog Norway; ^9^ Life and Health Sciences Research Institute (ICVS) University of Minho, Campus de Gualtar Braga Portugal; ^10^ ICVS/3B's—PT Government Associate Laboratory Braga Portugal; ^11^ Laboratory of Medical Investigation (LIM14), Faculty of Medicine University of São Paulo São Paulo Brazil

**Keywords:** Anyplex II HPV assay, cobas 4800 HPV test, HPV screening, human papillomavirus, next generation sequencing, performance, polymorphism, quality assurance

## Abstract

Human papillomavirus (HPV) genotype classification relies on DNA sequence similarity to reference (prototype) sequences. Most HPV assays used for cervical cancer screening were clinically validated against European HPV prototypes. However, the impact of HPV sequence polymorphisms on test performance remains unexplored. We evaluated whether sequence variation in E6/E7 relative to HPV prototypes affects test performance by analyzing cervicovaginal samples from 990 women enrolled (2019‐2022) across Belgium, Portugal, Brazil and Ecuador. Samples were tested using cobas, Anyplex, and next‐generation sequencing (Ampliseq/Ion Torrent targeting E6/E7). Sequence variation was defined as the proportion of single nucleotide polymorphisms across E6/E7 relative to reference sequence. Sequence variation was, on average, higher in HPV‐negative than HPV‐positive samples for HPV16 (0.46% vs 0.13%) and HPV18 (0.44% vs 0.37%) using cobas. Similar patterns were observed with Anyplex (HPV16: 0.78% vs 0.13%, HPV33: 0.66% vs 0.40%, HPV58: 0.79% vs 0.53, and HPV66: 1.14% vs 0.25%). For HPV45, sequence variation was, on average, higher in HPV‐positive than HPV‐negative samples when tested with Anyplex (0.87% vs 0.43%). For HPV types 18, 31, 35, 39, 51, 52, 56, 59 and 68, the mean sequence variation was similar between HPV‐negative and HPV‐positive samples using Anyplex. Our findings show that sequence variation relative to prototypes may impact test performance.

## 1 Introduction

Infection with human papillomavirus (HPV) is the primary cause of nearly all cases of cervical cancer [1]. The HPV genome comprises seven early (E) genes and two late (L) genes, with E6, E7, and L1 being the most extensively studied due to their key roles in HPV's pathogenicity and detection [1, 2]. The E6 and E7 oncoproteins drive HPV‐induced carcinogenesis by inactivating key tumor suppressors, primarily p53 and Rb, thus disrupting cell cycle regulation. The L1 gene, being highly conserved and commonly used for HPV classification, is targeted in diagnostic assays [1, 2]. Although more than 200 HPV types have been identified, only about 13 (HPVs 16, 18, 31, 33, 35, 39, 45, 51, 52, 56, 58, 59 and 68) are considered oncogenic based on their strong association with cervical cancer. The classification and nomenclature of these HPV types are overseen by the International HPV Reference Center (IHRC), which manages the taxonomy of HPV at the type level (i.e., below species level). The IHRC is responsible for confirming DNA sequences of novel HPV types and maintaining the corresponding reference clones, known as prototypes, used for molecular and clinical research [3]. Sequencing of HPV prototype genomes began in the 1980s, starting with HPV1 and later extended to high‐risk types like HPV16, which was sequenced by a German research group [4]. This same group also sequenced HPV18, originally isolated from a Brazilian patient [5]. Over the years, additional HPV types (35, 39, 45, 52, 56, and 66) were identified and sequenced, primarily by this group. Other research teams contributed to the sequencing of HPVs 31, 51, and 68 in the USA, HPV58 in Japan, and HPV59 in Korea. HPV68, sequenced in 2005, was the last high‐risk HPV (hrHPV) type added to the reference clone repository. Since then, no new oncogenic HPV types have been identified, although all reference sequences have been revised over time to ensure accuracy [6].

The PapillomaVirus Episteme (PaVE) was established to provide the scientific community with a highly organized, curated, and accessible papillomavirus genomics information and tools. PaVe includes curated sequences from both officially recognized types and 254 non‐reference HPV genomes that have not been formally validated by the IHRC [6]. Many of the original HPV sequences submitted to GenBank and RefSeq contained errors. These records have been corrected and updated through submissions to the Los Alamos Papillomavirus resource, based on the rationale that if these mutations or errors are not found in multiple variants of the same HPV type, they likely reflect sequencing errors. As a result, revised Reference Genomes, referred to as HPV_REF, have been made available for download via PaVe. These revised HPV reference sequences have been instrumental in the development of numerous commercial HPV diagnostic assays, including the cobas 4800 HPV Test (Roche Diagnostics) (cobas for short henceforward) and Anyplex II HPV HR detection (Seegene) (Anyplex for short henceforward). Both tests are clinically validated [7] and designed to detect 14 hrHPV types: 16, 18, 31, 33, 35, 39, 45, 51, 52, 56, 58, 59, 66, and 68. While HPV66 is no longer considered a hrHPV type due to its negligible cancer‐attributable fraction, it was previously classified as possibly oncogenic, hence many assays developed during that time (including current commercial ones) continue to include this genotype [7].

The cobas test targets the L1 region, individually genotyping HPV16 and HPV18, while detecting the remaining 12 hrHPV types as a pooled result. It provides cycle threshold (Ct) values, which are not a direct measure of viral load but can serve as an approximate proxy, with lower Ct values generally indicating higher levels of viral DNA. The cutoffs to consider a sample as positive are: Ct ≤ 40.50 for HPV16 and < 40.00 for HPV18 and the pooled hrHPV types. In contrast, Anyplex targets genomic regions that are not publicly disclosed by the manufacturer, potentially extending beyond the L1 region. Unlike cobas, Anyplex detects all 14 hrHPVs individually and provides semiquantitative results based on signal strength – often interpreted as a proxy for viral load. The assay classifies signal strength into three categories: high (signal detected before 31 PCR cycles), medium (signal detected between 31 and 39 PCR cycles), and low (signal detected after 40 PCR cycles of the total 50 cycles) [8, 9]. In addition to these commercially available tests, next‐generation sequencing (NGS) has emerged as a powerful tool for HPV detection, allowing for the identification of multiple concurrent HPV infections and enabling in‐depth analysis of whole HPV genomes, including the detection of single nucleotide polymorphisms (SNPs), integration sites of HPV DNA within the human genome, and epigenetic modifications [10, 11, 12].

In this context, we compared the performance of HPV detection using the cobas and Anyplex assays against NGS, which targets the E6/E7 regions of the viral genome. We also evaluated the impact of cobas Ct values and Anyplex signal strength on E6/E7‐based HPV detection by NGS (E6/E7‐NGS for short henceforward). Additionally, we examined the extent of sequence variation in the E6/E7 regions – measured as as the proportion of single nucleotide polymorphisms (SNPs) across the E6/E7 region relative to the reference sequence– and assessed its potential impact on the diagnostic performance of both commercial assays. We hypothesized that higher sequence divergence in the E6/E7 region relative to reference prototypes could influence detection by commercial assays, particularly in samples from genetically diverse populations.

## 2 Materials and Methods

### 2.1 Study Population and Data Collection

We used data from the ELEVATE (EarLy dEtection of cerVical cAncer in hard‐to‐reach populations of women through portable and point‐of‐care HPV Testing) study, the details of which have been previously described [13, 14]. Conducted between November 2019 and August 2022, the study enrolled women attending routine cervical cancer screening or colposcopy clinics across four countries. Cervicovaginal samples were collected at Ghent University Hospital (Belgium), Instituto Portugues de Oncologia de Lisboa (Portugal), Barretos Cancer Hospital (Brazil) as well as at the Sociedad de Lucha contra el Càncer (SOLCA)(Ecuador). Eligible participants were sexually active, nonpregnant women. Those who were menstruating at the time of their visit or had a history of hysterectomy were excluded. Participants completed a paper‐based questionnaire on sociodemographic and behavioral characteristics; responses were later transcribed into an electronic database by research assistants. Cervicovaginal samples were collected by a gynecologist or nurse using two Viba‐Brush self‐testing devices (Rovers, Oss, The Netherlands), held together mimicking self‐sampling. The brushes were used to swab the cervix area (excluding the endocervical region) and part of the superficial vaginal wall. In Belgium, Portugal, and Brazil, the brushes were rinsed in a 20 ml of ThinPrep pap test medium (Hologic Inc., Mississauga, ON, Canada), while in Ecuador, they were rinsed in 20 ml of Roche cell collection medium (Roche Diagnostics, Indianapolis, IN). Samples were stored at 2°C–8°C pending transfer to testing laboratories. One sample was sent to Barretos Cancer Hospital in Brazil for HPV testing with cobas and E6/E7‐NGS detection, while the second sample was shipped to Ghent University Hospital in Belgium for testing using Anyplex.

Ethical approval for the study was obtained from the institutional review boards at Ghent University Hospital (reference 2019/1687), Cuenca University (reference 2018‐074EO‐I‐Ext#1), Health of Instituto Portugues de Oncologia de Lisboa Francisco Gentil (reference UIC/1267 and 290/021), and the National Research Ethics Commission of Brazil (reference 16983119.7.0000.5437). All participants provided a signed written informed consent.

### 2.2 HPV Genotyping by Cobas and Anyplex

The cobas and Anyplex platforms utilize real‐time polymerase chain reaction (qPCR) to amplify and detect HPV DNA, with the human β‐globin gene included as an internal control to verify sample adequacy. Samples stored at Barretos Cancer Hospital were tested by cobas between October 2020 and September 2022, using 1 mL of an aliquoted sample. Samples stored at Ghent University were tested by Anyplex between June and August 2023, using 300 µL aliquots. The Anyplex assay was performed on a CFX96 real‐time thermocycler (Bio‐Rad, Hercules, CA), with technicians blinded to cobas results. Both assays were performed in accordance with the respective manufacturer's instructions.

### 2.3 HPV Detection by NGS

A 2 ml aliquot was centrifuged at 8000 g for 10 min, after which the supernatant was discarded, leaving approximately 250 µL. The resulting pellet was resuspended in the remaining volume and 25 µL of Proteinase K was added. Samples were then incubated for 3 h and 30 min at 70 g. After incubation, DNA extraction was performed using the QIAamp® DNA Media Kit (Qiagen, Germany), following the manufacturer's protocol, with the vacuum steps replaced by centrifugation at 2000g for 3 min. At the end of the protocol, DNA was eluted in 50 µL of AVE, quantified using the Qubit platform and stored at −20°C, awaiting NGS testing. The ELEVATE Brazilian team at Barretos Cancer Hospital designed 129 pairs of primers, divided into 3 pools, to amplify the E6 and E7 regions of all 14 hrHPV types. Primers were specifically optimized for ultrahigh‐multiplex performance using Ion AmpliSeq™ technology, including proprietary chemical modifications that render them incompatible with conventional PCR. DNA library preparation was performed manually according to the Ion AmpliSeq™ Library Kit Plus user guide protocol at 5x concentration, using 25 cycles for the initial PCR. Template preparation was carried out on the Ion Chef System equipment ‐ Thermo Scientific, USA, and sequencing was performed on the Ion Torrent S5 equipment following the manufacturer's protocol. Two Ion 520™ chips were used per run, each processing 32 samples, with the run designed to achieve an average target depth of 1000× per sample. For further details on primer design, refer to the Data Availability section.

Bioinformatic analysis was carried out at the International Human Papillomavirus Reference Center (Karolinska Institute) and the Norwegian HPV Reference Laboratory, following a comprehensive analytical pipeline. First, BAM files were converted to FASTQ format using SamTool Bam2fq. The resulting FASTQ sequences were filtered with the Trimmomatic tool, applying a minimum quality score of 20, a sliding window of 4, and a minimum read length of 75 bp. Second, the filtered reads were mapped to the reference genomes of the 14 hrHPV types using Hisat2, with the analysis restricted to the E6/E7 regions. Reads mapping outside these regions were excluded from further analysis. Third, variant calling was conducted by comparing sample reads to the reference sequences, identifying single nucleotide variants (SNVs). Finally, sequences with a read depth greater than 100 and a mean quality score above 20 were retained for analysis, while bases with lower quality were marked as “N.” Samples in which more than 25% of sequences were marked as “N” were excluded from further analysis. Single‐nucleotide polymorphisms (SNPs) were defined as positions where the consensus base in the sample differed from the reference base. Sequence variation was quantified as the proportion of SNPs identified in the E6/E7 region of each HPV type, relative to the corresponding prototype reference sequence, with higher values indicating greater variation (i.e., lack of homology) from the prototype reference sequence. The total number of such differing positions was divided by the length (in base pairs) of the E6/E7 region to yield a percentage.

### 2.4 Statistical Analysis

We described the characteristics of the study population, summarized HPV positivity (for single and multiple infections) by each assay, and assessed the concordance between E6/E7‐NGS detection and HPV test results from cobas and Anyplex using Cohen's kappa statistics (κ). Concordance was evaluated overall (for any HPV type) and separately for HPV16, HPV18, and the 12 pooled hrHPV types. The range of Kappa values reflects the degree of agreement; these were interpreted as follows: 0–0.2 poor, 0.21–0.4 fair, 0.41–0.6 moderate, 0.61–0.8 good, and 0.81–1.0 excellent [15]. We summarized, according to E6/E7‐NGS detection results, the Ct values from the cobas assay and the semi‐quantitative signal strength from the Anyplex assay. Median Ct values were compared using the Mann–Whitney test, and trends in signal strength categories were evaluated using the Cochran–Armitage test. We compared sequence variation with HPV detection by cobas and Anyplex, and tested significance using the Mann–Whitney test. We also carried out a sensitivity analysis to explore whether results may differ by geographic region (Brazil/Ecuador vs Belgium/Portugal). We conducted all analyses using Stata BE v.18.0 (2023), with a *p*‐value < 0.05 considered statistically significant.

## 3 Results

**Figure 1:**
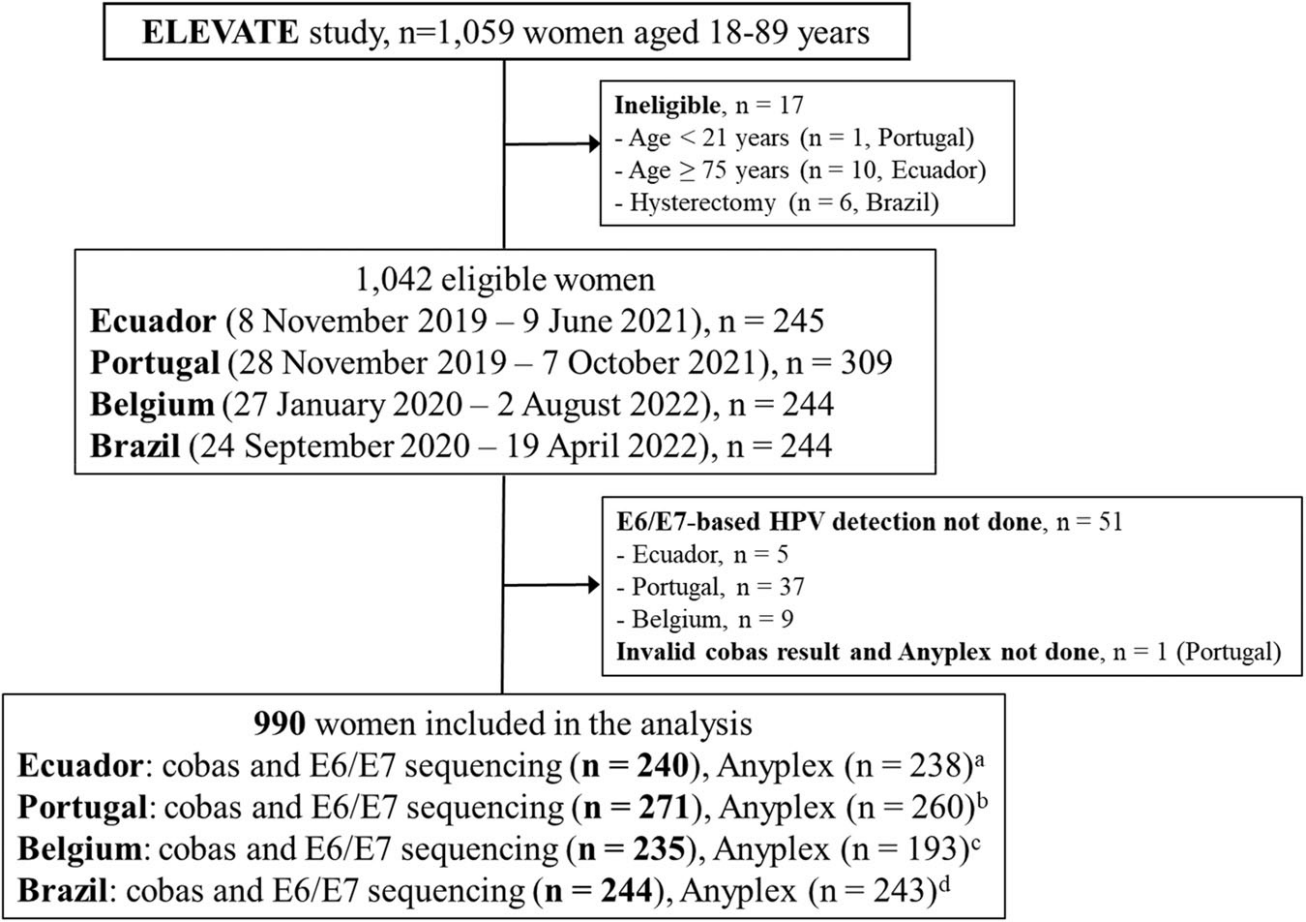
Overview of the study population and analytic sample. ^a^Two samples were depleted. ^b^11 samples were depleted. ^c^42 samples were depleted. ^d^One sample was depleted. ELEVATE, EarLy dEtection of cerVical cAncer in hard‐to‐reach populations of women through portable and point‐of‐care HPV TEsting; HPV, human papillomavirus.

The study included a total of 990 women, with sample sizes varying across countries and test types, as detailed in Figure 1. Participant ages ranged from 21.6 to 74.3 years, with a mean age of 44.8 years (SD = 10.1) and a median of 43.9 years (IQR = 15.3). Supplementary Table 1 outlines the participants' demographic characteristics. Most participants had attended university (28.4%) or completed secondary education (25.7%), with marked differences by geographic region (Brazil/Ecuador vs Belgium/Portugal). Nearly half of the participants were in a relationship or married (48.6%), while 17.8% were single and 11.5% were divorced. The majority were born in South America or the Caribbean (51.2%), followed by Europe (27.2%). Missing data for age, education, marital status, and region of birth were consistent at approximately 18.5% across participants. Ethnicity was self‐reported, with the largest proportion identifying as Brown/Mixed race (33.6%), followed by White (32.1%). Fewer participants identified as Indigenous (0.6%), Black (2.5%), or Asian (1.2%). Notably, ethnicity data was not collected in Portugal, which accounts for most of the missing data in this category.

Around 49% of all participants tested positive for at least one HPV type (47.8% by cobas and 50.1% by Anyplex). HPV16 was detected in 14.0% of participants 14.0% by cobas and in 14.9% by Anyplex, while HPV18 was identified in 3.7% by cobas and in 3.8% by Anyplex. Detection of other hrHPVs occurred in 36% of women tested by cobas and in 37.9% tested by Anyplex. Most HPV16 and HPV18 constituted single infections, as detected by both cobas and E6/E7‐NGS (Supplementary Table 2). Similarly, single infections – for the individual HPV types tested for by Anyplex and E6/E7‐NGS – counted for ~70% of the samples (Supplementary Table 3), with multiple infections being less frequent and typically involving two genotypes.

As shown in Table 1, E6/E7‐NGS HPV detection demonstrated good to excellent concordance with both cobas (κ ranging from 0.74 to 0.87) and Anyplex (κ ranging from 0.71 to 0.84) results. All κ values were statistically significant (*p* < 0.001), indicating nonrandom agreement between methods. Slightly higher concordance was observed between E6/E7‐NGS detection and cobas than with Anyplex across all HPV comparisons.

**Table 1:** Concordance between E6/E7‐NGS HPV detection and HPV test results by cobas and Anyplex^a^.

High‐risk HPV	HPV E6/E7‐NGS	cobas, *n* (%)^a^			Anyplex, *n* (%)^b^		
		+	−	Kappa	+	−	Kappa
Any HPV^c^	**+**	446 (45.1)	96 (9.7)	0.7546	426 (45.6)	86 (9.2)	0.7259
	**−**	26 (2.6)	422 (42.6)		42 (4.5)	380 (40.7)	
HPV16	**−**	126 (12.7)	18 (1.8)	0.8722	120 (12.8)	19 (2.0)	0.8357
	**−**	13 (1.3)	833 (84.1)		20 (2.1)	775 (83.0)	
HPV18	**+**	29 (2.9)	4 (0.4)	0.8223	26 (2.8)	3 (0.3)	0.8059
	**−**	8 (0.8)	949 (95.9)		9 (1.0)	896 (95.9)	
12 pooled HPVs^d^	**+**	328 (33.1)	92 (9.3)	0.7448	309 (33.1)	85 (9.1)	0.7107
	**−**	29 (2.9)	541 (54.6)		45 (4.8)	495 (53.0)	

*Note:* All Kappa values were statistically significant (*p* < 0.001).

Abbreviation: HPV, human papillomavirus.

^a^ Includes samples (*n* = 990) that had an HPV result by cobas and next generation sequencing.

^b^ Includes samples (*n* = 934) that had an HPV result by Anyplex and next generation sequencing.

^c^ Positivity to any of HPVs 16, 18, 31, 33, 35, 39, 45, 51, 52, 56, 58, 59, 66, and/or 68.

^d^ Includes HPVs 31, 33, 35, 39, 45, 51, 52, 56, 58, 59, 66, and 68.

Analysis of cobas Ct values (Table 2) showed that, among HPV‐positive samples, those testing negative by E6/E7‐NGS detection had significantly higher median Ct values compared to E6/E7‐NGS positive samples. This pattern was consistent across all HPV types and categories: HPV16 (37.2 vs. 29.1), HPV18 (37.3 vs. 30.4), and the pooled 12 hrHPVs (36.4 vs. 30.2). Samples with lower viral levels (reflected by higher Ct values) were more likely to be missed by E6/E7‐NGS detection. For Anyplex signal strength data (Table 3), a dose–response trend was observed: samples that were negative by E6/E7‐NGS generally had lower signal strengths compared to E6/E7‐NGS positive samples. This trend was statistically significant for most HPV types, except for HPVs 35, 56, 59, and 68, where the trend was present but did not reach significance (*p* > 0.05). Among these exceptions, all but HPV59 had fewer negative samples with high signal strength. Notably, for 9 out of 14 hrHPV types, none of the E6/E7‐NGS negative samples were classified with high signal strength by Anyplex.

**Table 2:** Distribution of Ct values for cobas according to E6/E7‐NGS HPV detection^a^.

HPV positivity by cobas	HPV E6/E7‐NGS	Ct values‐cobas			
		Range	Mean ± SD	Median (IQR)	*p* value^b^
**HPV16** *n* = 139	**+**	17.1–40.4	29.4 ± 4.6	29.1 (6.7)	0.0003
	**−**	22.5–40.4	35.1 ± 5.4	37.2 (5.1)	
**HPV18** *n* = 37	**+**	21.4–38.4	30.1 ± 5.3	30.4 (8.0)	0.0028
	**−**	32.3–38.2	36.1 ± 2.1	37.3 (2.9)	
**12 pooled HPVs** c *n* = 357	**+**	16.2–40.0	30.0 ± 5.3	30.2 (8.6)	0.0000
	**−**	16.6–40.0	35.0 ± 5.3	36.4 (5.7)	

Abbreviations: Ct, cycle threshold; HPV, human papillomavirus; IQR, interquartile range; SD, standard deviation.

^a^ Includes samples (*n* = 472) that had an HPV result by cobas and next generation sequencing and tested HPV positive by cobas for a given HPV type/pool.

^b^ For the Mann–Whitney test.

^c^ Includes HPVs 31, 33, 35, 39, 45, 51, 52, 56, 58, 59, 66, and 68.

**Table 3:** Distribution of signal strength for Anyplex according to E6/E7‐NGS HPV detection^a^.

HPV positivity by Anyplex	HPV E6/E7‐NGS	Signal strength‐Anyplex, *n* (%)			
		Low	Moderate	High	*p* value^b^
HPV16	**+**	11 (52.4)	75 (90.4)	34 (94.4)	0.0001
	**−**	10 (47.6)	8 (9.6)	2 (5.6)	
HPV18	**+**	3 (27.3)	15 (93.8)	8 (100.0)	0.0001
	**−**	8 (72.7)	1 (6.2)	0	
HPV31	**+**	10 (47.6)	43 (93.5)	6 (100.0)	0.0000
	**−**	11 (52.4)	3 (6.5)	0	
HPV33	**+**	1 (20.0)	11 (73.3)	5 (100.0)	0.0067
	**−**	4 (80.0)	4 (26.7)	0	
HPV35	**+**	2 (50.0)	17 (68.0)	6 (85.7)	0.2044
	**−**	2 (50.0)	8 (32.0)	1 (14.3)	
HPV39	**+**	1 (20.0)	12 (85.7)	4 (100.0)	0.0044
	**−**	4 (80.0)	2 (14.3)	0	
HPV45	**+**	4 (36.3)	16 (94.1)	4 (100.0)	0.0010
	**−**	7 (63.7)	1 (5.9)	0	
HPV51	**+**	6 (46.1)	16 (80.0)	9 (100.0)	0.0036
	**−**	7 (53.9)	4 (20.0)	0	
HPV52	**+**	12 (60.0)	20 (87.0)	4 (100.0)	0.0196
	**−**	8 (40.0)	3 (13.0)	0	
HPV56	**+**	12 (85.7)	12 (85.7)	5 (100.0)	0.4935
	**−**	2 (14.3)	2 (14.3)	0	
HPV58	**+**	4 (44.4)	21 (87.5)	16 (100.0)	0.0007
	**−**	5 (55.6)	3 (12.5)	0	
HPV59	**+**	7 (53.8)	17 (89.5)	10 (83.3)	0.0716
	**−**	6 (46.2)	2 (10.5)	2 (16.7)	
HPV66	**+**	6 (42.9)	19 (82.6)	10 (90.9)	0.0052
	**−**	8 (57.1)	4 (17.4)	1 (9.1)	
HPV68	**+**	7 (50.0)	9 (64.3)	2 (66.7)	0.4373
	**−**	7 (50.0)	5 (35.7)	1 (33.3)	

Abbreviation: HPV, human papillomavirus.

^a^ Includes samples (*n* = 468) that had an HPV result by Anyplex and next generation sequencing and tested HPV positive by Anyplex for a given HPV type.

^b^ For the Cochran–Armitage test.

Analysis of sequence variation in the E6/E7 region showed that samples detected by E6/E7‐NGS but not by cobas or Anyplex generally had a higher proportion of SNPs compared to those detected by these assays (Table 4). For HPV16, the median percentage of SNPs was significantly higher in samples that tested negative by cobas (0.46% vs. 0.13%) and negative by Anyplex (0.78% vs. 0.13%) compared to their respective positive counterparts. A similar trend was observed for HPV18 with cobas (0.44% vs. 0.37%), but the difference was not statistically significant. In contrast, for Anyplex, no difference in SNP proportions was observed between HPV18‐positive and ‐negative samples.

**Table 4:** Sequence variation relative to the HPV16 and HPV18 prototype sequences, based on E6/E7‐NGS detection, according to HPV test results by cobas and Anyplex.

HPV E6/E7‐NGS detection	HPV test	HPV test results	*n* (%)	Lack of homology, %^a^			
				Range	Median	Q1, Q3	*p* value^b^
HPV16	**cobas** c *n* = 144	**+**	126 (87.5)	0.00–1.46	0.13	0.00, 0.26	0.0328
		**−**	18 (12.5)	0.00–1.46	0.46	0.13, 1.19	
	**Anyplex** d *n* = 139	**+**	120 (86.3)	0.00–1.46	0.13	0.00, 0.20	0.0219
		**−**	19 (13.7)	0.00–1.33	0.80	0.13, 1.19	
HPV18	**cobas** c *n* = 33	**+**	29 (87.9)	0.00–1.62	0.37	0.37, 1.25	0.6687
		**−**	4 (12.1)	0.25–0.50	0.44	0.31, 0.50	
	**Anyplex** d *n* = 29	**+**	26 (89.7)	0.00–1.62	0.37	0.37, 1.25	0.2148
		**−**	3 (10.3)	0.25–0.37	0.37	0.25, 0.37	

*Note:* Sequence variation was defined as the proportion of single nucleotide polymorphisms across the E6/E7 region relative to the reference sequence.

Abbreviations: HPV, human papillomavirus; Q, quartile.

^a^ Calculated by dividing the number of polymorphisms by the length of E6/E7 gene *100.

^b^ For the Mann–Whitney test.

^c^ Includes samples that had an HPV result by cobas and next generation sequencing.

^d^ Includes samples that had an HPV result by Anyplex and next generation sequencing.

Table 5 shows that, compared to E6/E7‐NGS detection, samples that were negative by Anyplex had a significantly higher degree of sequence variation for HPV33 (0.66% vs. 0.40%), HPV58 (0.79% vs. 0.53%), and HPV66 (1.14% vs. 0.25%). For the remaining HPV types (18, 31, 35, 39, 51, 52, 56, 59, and 68), sequence variation was comparable between Anyplex‐positive and Anyplex‐negative samples. The only exception was for HPV45, where Anyplex‐positive samples had a higher sequence variation (0.87% vs. 0.43%), but this difference was not statistically significant likely due to the small number of Anyplex‐negative samples for this genotype (*n* = 2). As shown in Supplementary Tables 4 and 5, a similar pattern was observed upon stratifying the results by geographic region. For HPV16, samples from Brazil/Ecuador showed significantly higher sequence divergence in cobas‐ and Anyplex‐negative cases compared to their counterparts, and the majority of discordant samples originated from these countries. In contrast, no such difference was observed in Belgium/Portugal. For other hrHPV types, region‐specific trends were also evident, with greater divergence in negative samples for HPV66 and HPV68 in Latin America, and HPV33 and HPV58 in Europe. Differences for remaining genotypes were not statistically significant.

**Table 5:** Sequence variation relative to the prototype sequences of the 12 other high‐risk HPVs, based on E6/E7‐NGS detection, according to HPV test results by Anyplex.

HPV E6/E7‐NGS detection	HPV test results by Anyplex^b^	*n* (%)	Sequence diversity, %^a^			
			Range	Median	Q1, Q3	*p*‐value^c^
HPV31	**+**	59 (68.6)	0.00–1.33	1.20	0.40, 1.20	0.8876
	**−**	27 (31.4)	0.13–1.33	1.20	1.07, 1.20	
HPV33	**+**	17 (77.3)	0.00–1.58	0.40	0.00, 0.53	0.0379
	**−**	5 (22.7)	0.40–1.19	0.66	0.66, 0.66	
HPV35	**+**	25 (80.7)	0.27–0.80	0.67	0.67, 0.67	0.3049
	**−**	6 (19.4)	0.67–0.67	0.67	0.67, 0.67	
HPV39	**+**	17 (85.0)	0.00–0.12	0.00	0.00, 0.00	0.4418
	**−**	3 (15.0)	0.00–0.00	0.00	0.00, 0.00	
HPV45	**+**	24 (92.3)	0.00–1.49	0.87	0.00, 0.99	0.1343
	**−**	2 (7.7)	0.00–0.87	0.43	0.00, 0.87	
HPV51	**+**	31 (72.1)	0.00–0.78	0.00	0.00, 0.13	0.1331
	**−**	12 (27.9)	0.00–0.13	0.00	0.00, 0.13	
HPV52	**+**	36 (75.0)	0.00–1.20	0.13	0.00, 0.27	0.9098
	**−**	12 (25.0)	0.00–0.53	0.13	0.00, 0.27	
HPV56	**+**	29 (74.4)	0.00–0.76	0.51	0.51, 0.63	0.8638
	**−**	10 (25.6)	0.00–0.76	0.51	0.51, 0.51	
HPV58	**+**	41(80.4)	0.26–1.71	0.53	0.53, 0.53	0.0205
	**−**	10 (19.6)	0.53–1.18	0.79	0.53, 1.05	
HPV59	**+**	34 (60.7)	0.00–0.00	0.00	0.00, 0.00	—
	**−**	22 (39.3)	0.00–0.00	0.00	0.00, 0.00	
HPV66	**+**	35 (66.0)	0.00–1.52	0.25	0.00, 1.27	0.0323
	**−**	18 (34.0)	0.00–1.52	1.14	1.14, 1.27	
HPV68	**+**	18 (51.4)	0.00–0.12	0.00	0.00, 0.00	0.1373
	**−**	17 (48.6)	0.00–0.61	0.00	0.00, 0.12	

*Note:* Sequence variation was defined as the proportion of single nucleotide polymorphisms across the E6/E7 region relative to the reference sequence.

Abbreviation: HPV, human papillomavirus; Q, quartile.

^a^ Calculated by dividing the number of polymorphisms by the length of E6/E7 gene *100.

^b^ Included samples (*n* = 394) that had an HPV result by Anyplex and next generation sequencing.

^c^ For the Mann–Whitney test.

## 4 Discussion

In this study, we assessed the concordance between E6/E7‐based HPV detection via NGS and two widely used, clinically validated commercial assays: cobas and Anyplex [7]. We observed good overall agreement between E6/E7‐NGS and both assays for individual genotypes and pooled results, with slightly higher concordance between E6/E7‐NGS and cobas than with Anyplex. We also found that the E6/E7‐NGS method is more likely to miss samples with lower viral load, as reflected by the findings of significantly higher Ct values (cobas) and lower signal strength (Anyplex) in E6/E7‐NGS negative cases. This suggests that viral load plays a critical role in the sensitivity of E6/E7‐NGS detection, particularly at the lower limits of the assays' performance.

The superior ability of E6/E7‐NGS to detect more HPV‐positive samples than commercial assays could be explained by two main factors. First, the E6 and E7 genes are directly involved in HPV‐driven cellular transformation, whereas the L1 gene encodes the viral capsid protein [2]. That is, whereas the L1 gene can be lost during the viral integration process that occurs in more advanced stages of the disease, the E6 and E7 genes are typically retained [16]. Second, differences in detection techniques (i.e., NGS, qPCR, and TOCE PCR) can impact concordance and detection of hrHPV types [10]. A major challenge in comparing these methods arises from the lack of standardized guidelines for NGS analysis, resulting in considerable variability across platforms and research groups [10]. This variability stems from differences in NGS techniques (i.e., differences in sequencing platforms, library preparation methods, and targeted genomic regions) and especially in bioinformatics pipelines, with each applying distinct criteria for quality control, read mapping, and variant calling, further influencing the HPV detection results [17, 18, 19, 20, 21]. For instance, some studies prioritize high‐depth coverage, such as > 500× for sequencing [19], or a median depth of > 200× with at least 80% genome coverage and positions covered by ≥ 5 reads considered valid [17]. In contrast, other approaches, particularly those focused on mutation classification, may not explicitly define criteria for positive sample identification [21]. Additionally, thresholds such as a minimum read depth of 30 for genotype confirmation [20] or a minimum sequence identity of 90% for genotype assignment [18] illustrate the diversity in bioinformatics strategies. Our pipeline applied stringent thresholds for depth and quality, focusing specifically on the E6/E7 regions. While aligned with targeted, high‐quality approaches, this approach differs from genome‐wide or less transparent strategies. This variability underscores the importance of establishing harmonized guidelines to improve the comparability and reproducibility across HPV NGS studies.

We also found that a higher degree of sequence variation in E6/E7 correlated with an increased likelihood of HPV non‐detection by cobas and Anyplex– particularly for HPV16, HPV33, HPV58, and HPV66. This finding suggests that increased dissimilarity from the prototype HPV sequences may compromise test performance. Since cobas targets the L1, we hypothesize that sequence variation in E6/E7 may reflect similar variation in L1. Although Anyplex is often described as targeting L1, recent evidence from a related assay (Allplex HPV28) suggests amplification may also involve other genomic regions, including E6/E7 [22], which could partially mitigate this limitation. Specifically, HPV16‐positive samples with a higher degree of sequence variation were more frequently missed by these commercial tests, suggesting that their design may not fully capture the genetic diversity of HPV16 variants circulating in diverse populations.

This observation is consistent with the virus's evolutionary history, characterized by limited HPV recombination, resulting in a genetic drift over time [23, 24]. The phylogenetic similarity between HPV16/HPV18 and various human racial groups (Africans, Caucasians, Amerindians and East Asians) reinforces the idea of ancient coevolution, which likely contributed to the development of distinct viral lineages [5, 23]. These lineage‐specific differences raise important questions about whether current reference sequences sufficiently capture the full extent of HPV genetic diversity.

The PaVE database has been a key resource for HPV research, including in our study. While reference sequences based on the L1 region have long served as the foundation for HPV taxonomy, their utility, depending on the research/diagnostic aim, may be limited in capturing the broader genomic diversity of HPV strains [25, 26]. Currently, HPV taxonomy extends to the type‐level, with isolates of the same HPV type classified as variant lineages and sublineages if their complete genomes differ by approximately 1.0%−10.0% and 0.5%−1.0%, respectively. However, because sequence homology varies across genomic regions, full genome sequencing provides a more accurate classification than single‐gene analyses [1, 27, 28]. Recent studies have advocated for expanding reference resources and incorporating complementary strategies that better reflect HPV genomic diversity without altering the current taxonomic structure [25, 26]. For example, Trevino et al. proposed a de novo methodology for generating a reference database that includes a broader representation of HPV genomic diversity, extending beyond the L1 region, by using oligonucleotide frequency profiling and hierarchical clustering of whole‐genome sequences [29]. Their method successfully distinguished all 219 PaVE sequences. These findings suggest that future HPV classification systems could benefit from incorporating “synthetic references”, which could better reflect the complete genomic landscape of HPV, particularly as advanced genomic techniques become more common. Any revised classification should incorporate the complete variability of the HPV genome to ensure that reference databases like PaVE accurately represent the diversity of HPV sequences [29].

In addition to reference sequence limitations, population diversity must also be considered when evaluating HPV test performance. While updated genomic databases are essential for capturing viral diversity, many commercial HPV assays have been validated in demographically narrow cohorts, which may limit their accuracy in more diverse populations. These tests have primarily been validated in predominantly Caucasian populations, potentially overlooking genetic variations in other groups, such as those in Latin America [23, 29], and leading to diagnostic discrepancies. For example, the ATHENA study, which served as the basis for validating cobas for primary cervical cancer screening, included a homogenous sample of over 40,000 women in the United States, 83% of whom were white [30, 31, 32]. Similarly, Anyplex has not been extensively validated in ethnically diverse populations. The largest study, VALGENT‐3, included 1,600 patients primarily from Slovenia and compared the clinical and analytical performance of Anyplex with the Hybrid Capture 2 HPV DNA test (HC2) and cobas. Anyplex consistently demonstrated excellent clinical performance, supporting its validation for primary cervical cancer screening [33]. Another study in Korea (*n* = 1137) also found comparable performance between Anyplex and HC2 [34]. However, the underrepresentation of diverse populations in these validation studies raises concerns about the generalizability of test performance outside Europe and North America. This concern is particularly relevant in our study, where nearly half of the samples originated from Latin America.

Addressing this gap, our study included women from Ecuador, Brazil, Portugal, and Belgium, providing insights into how genetic diversity may influence the performance of HPV tests. Notably, Ecuador, a country with a high incidence of cervical cancer [35], has a unique population structure: 77% of individuals identify as mestizo (a mix of Indigenous, European and African ancestry), and 7.7% as Indigenous [36, 37]. This genetic heterogeneity may contribute to the circulation of a broader and potentially underrepresented range of variants that are not well detected by commercial assays predominantly validated in more genetically homogeneous populations. In our study (Supplementary Table 1), most participants self‐identified as of mixed ancestry (33.6%) or white (32.1%). However, in countries like Brazil and Ecuador, “white” identity often encompasses admixed genetic backgrounds. Although the ESTAMPA study is advancing our understanding of HPV screening in Latin America, it does not include Ecuador, highlighting the need for broader regional inclusion in HPV validation studies [38]. Collectively, our findings support the need to validate HPV assays in genetically diverse populations and to further investigate the potential impact of genetic variability on HPV test performance, particularly in regions with high cervical cancer burden. Consistent with this, our stratified results show that sequence divergence was significantly higher in undetected samples, particularly in Brazil and Ecuador for HPV16, HPV66, and HPV68, and in Belgium and Portugal for HPV33 and HPV58. These region‐specific trends support the idea that circulating variants may harbor polymorphisms in E6/E7 that affect molecular detection. Together, our findings reinforce the need to incorporate broader variant diversity into assay design and validation, especially in underrepresented populations.

As previously mentioned, a key limitation of our study was the exclusive amplification of the E6/E7 regions, which prevented direct assessment of the impact of polymorphisms in the L1 region on test performance. Notwithstanding, we infer that E6/E7 polymorphisms likely influence the L1 region, affecting the detection capability of commercial assays. For Anyplex, which may also target E6/E7 regions [22], this limitation might be partially mitigated, as its detection capability could be directly influenced by E6/E7 sequence variability. Furthermore, whole‐genome sequencing would have enabled more precise classification of viral lineages, allowing us to determine whether specific evolutionary lineages are more likely to evade detection by commercial assays. This would have provided a more comprehensive understanding of genotype and lineage‐specific detection patterns. Additionally, the relatively limited number of type‐specific HPV positive samples per country constrained our ability to perform stratified analyses by individual country. Another factor that may influence test performance is the occurrence of multiple HPV infections, particularly in assays that provide partial (cobas) or extended (Anyplex and NGS) genotyping. In our study, single infections were more frequently detected than multiple infections, thus minimizing any potential confounding by the presence of co‐infections. We therefore focused on each hrHPV type separately to isolate the impact of sequence divergence on detection.

Although our study focused on analytical performance, the under‐detection of certain hrHPV variants by commercial assays may have clinical implications, potentially leading to false‐negative results. If such variants are also associated with oncogenic potential, their missed detection may impact the efficacy of cervical cancer screening. While previous studies have shown comparable clinical performance between assays targeting L1 and E6/E7 regions, such as the Aptima mRNA‐based assay [39], our findings suggest that even small DNA‐level sequence variations in E6/E7 may compromise detection by molecular assays, particularly in genetically diverse populations. Further studies are needed to investigate whether these SNP are linked to persistence or lesion development.

In conclusion, our findings highlight the impact of viral genetic variability on the performance of commercial HPV tests, particularly in non‐European populations. Discrepancies in HPV detection, especially for HPV16, underscore the limitations of assays largely validated on A1 (formerly European) and A2‐A3 (formerly The Americas) HPV prototypes. These findings underscore the need to validate HPV tests in genetically diverse populations, especially in low‐ and middle‐income countries where the cervical cancer burden is greatest and non‐A sublineages are most prevalent [40]. Expanding validation efforts to include a broader spectrum of genetic diversity, alongside implementing standardized guidelines for NGS bioinformatics pipelines, will enhance the reliability of HPV detection and improve the effectiveness of global cervical cancer screening initiatives. Future research should focus on designing adaptable HPV testing methods that account for different genetic backgrounds, ensuring accurate, inclusive, and equitable screening worldwide.

## Author Contributions

Elizaveta Padalko, Bo Verberckmoes, Olivier Degomme, Heleen Vemandere, and Adhemar Longatto‐Filho contributed to the conceptualization of the ELEVATE study. Luani R. Godoy, Marian El‐Zein, and Eduardo L. Franco conceived and designed this sub‐study and secondary analyses. Luani R. Godoy, and Piet Cools participated in data collection. Luani R. Godoy performed NGS workflow steps. Luani R. Godoy, Laila Sara Arroyo Mühr, and Milan S. Stosic performed the bioinformatic analyses. Luani R. Godoy performed the statistical analyses, interpreted the data, tabulated the study results, and drafted the manuscript under the supervision of Marian El‐Zein and Eduardo L. Franco. All authors critically reviewed the manuscript and approved the final submitted version.

## Ethics Statement

The study received ethical approval by institutional review boards at Ghent University Hospital (reference 2019/1687), Cuenca University (reference 2018‐074EO‐I‐Ext#1), Health of Instituto Portugues de Oncologia de Lisboa Francisco Gentil (reference UIC/1267 and 290/021), and the National Research Ethics Commission of Brazil (reference 16983119.7.0000.5437). Participants provided written informed consent for participation in the study.

## Conflicts of Interest

E.L.F. has occasionally served as an advisor for companies involved in HPV vaccines (Merck, GSK) and HPV diagnostics (Roche Diagnostics). E.L.F.'s institution has received funding from Merck for investigator‐initiated studies in his laboratory. M.Z. and E.L.F. also hold a patent for the discovery titled “DNA methylation markers for early detection of cervical cancer”, registered with the Office of Innovation and Partnerships, McGill University, Montreal, Quebec, Canada (October 2018).

## Supporting information


**Supplementary Table 1**: Characteristics of the study population, overall and by geographic region. **Supplementary Table 2**: HPV positivity by cobas and E6/E7‐NGS, considering detection of single and multiple HPV infections. **Supplementary Table 3**: HPV positivity by Anyplex and E6/E7‐NGS, considering detection of single and multiple HPV infections. **Supplementary Table 4**: Sequence variation relative to the HPV16 and HPV18 prototype sequences, based on E6/E7‐NGS detection, according to HPV test results by cobas and stratified by geographic region (Brazil/Ecuador vs. Belgium/Portugal). **Supplementary Table 5**: Sequence variation relative to the prototype sequences of the 12 other high‐risk HPVs, based on E6/E7‐NGS detection, according to HPV test results by Anyplex and stratified by geographic region (Brazil/Ecuador vs. Belgium/Portugal).

## Data Availability

The data that support the findings of this study are available on request from the corresponding author. The data are not publicly available due to privacy or ethical restrictions. The data generated and analyzed during this study, along with the scripts used for statistical analysis, are available upon reasonable request from the ELEVATE consortium. The primers used were custom‐designed for the Ion AmpliSeq™ platform and include proprietary chemical modifications required for ultrahigh‐multiplex reactions. Due to these technical characteristics, they are not functional outside of the AmpliSeq™ system and cannot be reproduced or used with standard PCR protocols. As such, the full sequences are not publicly available. Further information may be provided upon request.
