# Supplementary material for: E6/E7 Similarity to Human Papillomavirus Prototypes and Performance of HPV Testing by Cobas 4800 HPV Test and Anyplex II HPV HR
Source: J Med Virol. 2025 Oct 28;97(11):e70668. doi: 10.1002/jmv.70668 (PMC12560617; doi:10.1002/jmv.70668)
Supplement: Supplementary file 1 — Supplementary Table 1: Characteristics of the study population, overall and by geographic region. Supplementary Table 2: HPV positivity by cobas and E6/E7‐NGS, considering detection of single and multiple HPV infections. Supplementary Table 3: HPV positivity by Anyplex and E6/E7‐NGS, considering detection of single and multiple HPV infections. Supplementary Table 4: Sequence variation relative to the HPV16 and HPV18 prototype sequences, based on E6/E7‐NGS detection, according to HPV test results by cobas and stratified by geographic region (Brazil/Ecuador vs. Belgium/Portugal). Supplementary Table 5: Sequence variation relative to the prototype sequences of the 12 other high‐risk HPVs, based on E6/E7‐NGS detection, according to HPV test results by Anyplex and stratified by geographic region (Brazil/Ecuador vs. Belgium/Portugal). [file JMV-97-e70668-s001.docx]

## **E6/E7 similarity to human papillomavirus prototypes and performance of HPV testing by cobas 4800 HPV test and Anyplex II HPV HR**

Luani R. Godoy,^a,b*^ Mariam El-Zein,^a*^ Piet Cools,^c^ Elizaveta Padalko,^d^ Bo Verberckmoes, ^e^ Olivier Degomme,^e^ Heleen Vermandere, ^e^ Laila Sara Arroyo Mühr,^f^ Milan S. Stosic,^g,h^ Eduardo L. Franco,^a^ Adhemar Longatto-Filho,^b,i,j,k^ and the ELEVATE study group^l^

**Supplementary Table 1**: Characteristics of the study population, overall and by geographic region.

| **Variables** | | **Overall, n=990** | | | **Brazil/Ecuador, n=484** | | **Belgium/Portugal, n=506** | |  |
| --- | --- | --- | --- | --- | --- | --- | --- | --- | --- |
| **Age**, years | | |  |  | |  | |  |  |
|  | Mean ± SD | 44.8 ± 10.1 | | | 46.3 ± 9.9 | | 43.4 ± 10.1 | |  |
|  | Median (IQR) | 43.9 (15.3) | | | 45.6 (14.8) | | 42.3 (15.1) | |  |
|  | Range | 21.6-74.3 | | | 24.3-74.3 | | 21-6-72.0 | |  |
| **Education, n (%)** | | |  |  | |  | |  |  |
|  | Pre-elementary | 97 (9.8) | | | 84 (17.3) | | 13 (2.6) | |  |
|  | Primary | 176 (17.8) | | | 142 (29.3) | | 34 (6.7) | |  |
|  | Secondary | 254 (25.7) | | | 169 (34.9) | | 85 (16.8) | |  |
|  | University | 281 (28.4) | | | 89 (18.4) | | 192 (37.9) | |  |
|  | *Missing* | 182 (18.4) | | | 0 (0.0) | | 182 (36.0) | |  |
| **Marital status, n (%)** | | |  |  | |  | |  |  |
|  | Single | 176 (17.8) | | | 104 (21.5) | | 72 (14.2) | |  |
|  | In a relationship/married | 481 (48.6) | | | 272 (56.2) | | 209 (41.3) | |  |
|  | Divorced | 114 (11.5) | | | 76 (15.7) | | 38 (7.5) | |  |
|  | Widow | 36 (3.6) | | | 32 (6.6) | | 4 (0.8) | |  |
|  | *Missing* | 183 (18.5) | | | 0 (0.0) | | 183 (36.2) | |  |
| **Region of birth, n (%)** | | |  |  | |  | |  |  |
|  | South America/Caribbean | 507 (51.2) | | | 484 (100.0) | | 23 (4.5) | |  |
|  | Europe | 269 (27.2) | | | 0 (0.0) | | 269 (53.2) | |  |
|  | Africa | 14 (1.4) | | | 0 (0.0) | | 14 (2.8) | |  |
|  | Asia | 16 (1.6) | | | 0 (0.0) | | 16 (3.2) | |  |
|  | *Missing* | 184 (18.6) | | | 0 (0.0) | | 184 (36.4) | |  |
| **Ethnicity, n (%)** | | |  |  | |  | |  |  |
|  | White | 318 (32.1) | | | 118 (24.4) | | 200 (39.5) | |  |
|  | Mixed ancestry | 333 (33.6) | | | 330 (68.2) | | 3 (0.6) | |  |
|  | Indigenous | 6 (0.6) | | | 6 (1.2) | | 0 (0.0) | |  |
|  | Black | 25 (2.5) | | | 22 (4.5) | | 3 (0.6) | |  |
|  | Asian | 12 (1.2) | | | 7 (1.4) | | 5 (1.0) | |  |
|  | *Missing* | 296 (29.9) | | | 1 (0.2) | | 295 (58.3) | |  |

IQR, interquartile range; SD, standard deviation

**Supplementary Table 2**: HPV positivity by cobas and E6/E7-NGS, considering detection of single and multiple HPV infections ^a^

| **HPV types** | | **HPV positivity by cobas,**  **n=472** | **HPV positivity by HPV E6/E7-NGS, n=542** |
| --- | --- | --- | --- |
| **Single infections**^b^ | **HPV16** | 93 | 100 |
|  | **HPV18** | 20 | 21 |
| **12 pooled HPVs**^d^ | **12 pooled HPVs^e^** | 302 | 368 |
| **Multiple infections**^c^ | **HPV16 and HPV18** | 2 | 1 |
|  | **HPV16 and 12 pooled HPVs^e^** | 40 | 41 |
|  | **HPV18 and 12 pooled HPVs^e^** | 11 | 9 |
|  | **HPV16, HPV18 and 12 pooled HPVs^d^** | 4 | 2 |

^a^ Includes samples that had an HPV result by both tests.

^b^ Includes samples that had an HPV result by both tests and are positive for only one HPV type by Anyplex.

^c^ Includes samples that had an HPV result by both tests and are positive for ≥2 HPV types by Anyplex.

^d^ Results classified as “12 pooled HPVs” do not discriminate between single and multiple infections.

^e^ Includes HPVs 31, 33, 35, 39, 45, 51, 52, 56, 58, 59, 66, and 68.

**Supplementary Table 3**: HPV positivity by Anyplex and E6/E7-NGS, considering detection of single and multiple HPV infections

| **HPV types** | | **HPV positivity by Anyplex, n=468** | **HPV positivity by E6/E7-NGS, n=512** |
| --- | --- | --- | --- |
| **Single infections**^a^ | HPV16 | 94 | 98 |
|  | HPV18 | 18 | 19 |
|  | HPV31 | 36 | 55 |
|  | HPV33 | 14 | 19 |
|  | HPV35 | 16 | 19 |
|  | HPV39 | 10 | 10 |
|  | HPV45 | 18 | 14 |
|  | HPV51 | 16 | 17 |
|  | HPV52 | 23 | 21 |
|  | HPV56 | 6 | 12 |
|  | HPV58 | 26 | 27 |
|  | HPV59 | 23 | 37 |
|  | HPV66 | 14 | 22 |
|  | HPV68 | 17 | 11 |
| **Multiple infections**^b^ | HPVs 16 and 18 | 2 | 1 |
|  | HPVs 16 and 31 | 4 | 4 |
|  | HPVs 16 and 33 | 2 | 1 |
|  | HPVs 16 and 35 | 1 | 1 |
|  | HPVs 16 and 39 | 1 | 1 |
|  | HPVs 16 and 45 | 2 | 4 |
|  | HPVs 16 and 51 | 3 | 3 |
|  | HPVs 16 and 52 | 1 | 1 |
|  | HPVs 16 and 56 | 2 | 2 |
|  | HPVs 16 and 58 | 1 | 1 |
|  | HPVs 16 and 59 | 4 | 5 |
|  | HPVs 16 and 66 | 4 | 2 |
|  | HPVs 16 and 68 | 1 | 3 |
|  | HPVs 18 and 31 | 1 | 0 |
|  | HPVs 18 and 35 | 0 | 1 |
|  | HPVs 18 and 39 | 1 | 0 |
|  | HPVs 18 and 51 | 1 | 0 |
|  | HPVs 18 and 52 | 1 | 1 |
|  | HPVs 18 and 56 | 1 | 1 |
|  | HPVs 18 and 58 | 0 | 1 |
|  | HPVs 18 and 66 | 1 | 0 |
|  | HPVs 18 and 68 | 1 | 1 |
|  | HPVs 31 and 33 | 1 | 1 |
|  | HPVs 31 and 35 | 1 | 2 |
|  | HPVs 31 and 45 | 2 | 1 |
|  | HPVs 31 and 51 | 1 | 0 |
|  | HPVs 31 and 52 | 1 | 0 |
|  | HPVs 31 and 56 | 2 | 1 |
|  | HPVs 31 and 58 | 1 | 0 |
|  | HPVs 31 and 59 | 2 | 3 |
|  | HPVs 31 and 66 | 4 | 6 |
|  | HPVs 31 and 68 | 1 | 1 |
|  | HPVs 33 and 58 | 1 | 0 |
|  | HPVs 33 and 59 | 1 | 0 |
|  | HPVs 33 and 66 | 2 | 0 |
|  | HPVs 35 and 45 | 1 | 1 |
|  | HPVs 35 and 51 | 1 | 0 |
|  | HPVs 35 and 52 | 1 | 0 |
|  | HPVs 35 and 56 | 4 | 3 |
|  | HPVs 35 and 58 | 1 | 1 |
|  | HPVs 35 and 59 | 1 | 0 |
|  | HPVs 35 and 66 | 1 | 1 |
|  | HPVs 39 and 45 | 1 | 0 |
|  | HPVs 39 and 51 | 2 | 1 |
|  | HPVs 39 and 52 | 0 | 1 |
|  | HPVs 39 and 58 | 1 | 0 |
|  | HPVs 39 and 59 | 1 | 0 |
|  | HPVs 39 and 66 | 1 | 2 |
|  | HPVs 45 and 56 | 1 | 0 |
|  | HPVs 45 and 58 | 0 | 1 |
|  | HPVs 45 and 59 | 0 | 1 |
|  | HPVs 45 and 66 | 1 | 0 |
|  | HPVs 51 and 52 | 1 | 1 |
|  | HPVs 51 and 56 | 1 | 2 |
|  | HPVs 51 and 58 | 1 | 2 |
|  | HPVs 51 and 66 | 2 | 3 |
|  | HPVs 51 and 68 | 2 | 8 |
|  | HPVs 52 and 56 | 0 | 2 |
|  | HPVs 52 and 58 | 4 | 4 |
|  | HPVs 52 and 59 | 2 | 2 |
|  | HPVs 52 and 66 | 1 | 3 |
|  | HPVs 56 and 58 | 1 | 1 |
|  | HPVs 56 and 59 | 1 | 1 |
|  | HPVs 56 and 66 | 2 | 3 |
|  | HPVs 56 and 68 | 0 | 1 |
|  | HPVs 58 and 59 | 0 | 1 |
|  | HPVs 58 and 66 | 1 | 0 |
|  | HPVs 58 and 68 | 0 | 2 |
|  | HPVs 59 and 66 | 1 | 0 |
|  | HPVs 59 and 68 | 2 | 1 |
|  | HPVs 66 and 68 | 1 | 1 |
|  | HPVs 16, 18, and 51 | 1 | 1 |
|  | HPVs 16, 31, and 35 | 1 | 0 |
|  | HPVs 16, 31, and 39 | 0 | 1 |
|  | HPVs 16, 31, and 51 | 1 | 0 |
|  | HPVs 16, 31, and 52 | 1 | 0 |
|  | HPVs 16, 31, and 59 | 1 | 1 |
|  | HPVs 16, 33, and 66 | 1 | 0 |
|  | HPVs 16, 39, and 68 | 1 | 1 |
|  | HPVs 16, 45, and 58 | 2 | 1 |
|  | HPVs 16, 51, and 56 | 0 | 1 |
|  | HPVs 16, 51, and 58 | 1 | 0 |
|  | HPVs 16, 51, and 59 | 0 | 1 |
|  | HPVs 16, 52, and 59 | 0 | 1 |
|  | HPVs 16, 56, and 66 | 0 | 1 |
|  | HPVs 16, 58, and 66 | 1 | 0 |
|  | HPVs 16, 58, and 68 | 1 | 1 |
|  | HPVs 16, 59, and 66 | 0 | 1 |
|  | HPVs 18, 33, and 35 | 1 | 0 |
|  | HPVs 18, 35, and 52 | 0 | 1 |
|  | HPVs 18, 39, and 52 | 1 | 1 |
|  | HPVs 18, 52, and 58 | 1 | 0 |
|  | HPVs 18, 58, and 59 | 0 | 1 |
|  | HPVs 31, 33, and 51 | 0 | 1 |
|  | HPVs 31, 39, and 51 | 1 | 0 |
|  | HPVs 31, 39, and 52 | 0 | 1 |
|  | HPVs 31, 51, and 59 | 1 | 0 |
|  | HPVs 31, 52, and 56 | 1 | 2 |
|  | HPVs 31, 52, and 68 | 0 | 1 |
|  | HPVs 31, 56, and 66 | 1 | 0 |
|  | HPVs 31, 56, and 68 | 1 | 0 |
|  | HPVs 31, 58, and 66 | 2 | 4 |
|  | HPVs 31, 59, and 68 | 1 | 0 |
|  | HPVs 33, 35, and 66 | 1 | 0 |
|  | HPVs 33, 52, and 66 | 1 | 0 |
|  | HPVs 35, 52, and 66 | 1 | 0 |
|  | HPVs 35, 56, and 68 | 1 | 0 |
|  | HPVs 39, 52, and 66 | 0 | 1 |
|  | HPVs 45, 52, and 56 | 2 | 1 |
|  | HPVs 45, 58, and 68 | 0 | 1 |
|  | HPVs 51, 56, and 68 | 0 | 1 |
|  | HPVs 52, 56, and 66 | 0 | 1 |
|  | HPVs 52, 66, and 68 | 0 | 1 |
|  | HPVs 16, 18, 35, and 52 | 1 | 0 |
|  | HPVs 16, 18, 56, and 66 | 1 | 0 |
|  | HPVs 16, 31, 51, and 56 | 1 | 0 |
|  | HPVs 16, 31, 51, and 59 | 1 | 0 |
|  | HPVs 16, 31, 56, and 58 | 1 | 1 |
|  | HPVs 16, 39, 45, and 66 | 1 | 0 |
|  | HPVs 18, 35, 51, and 52 | 1 | 0 |
|  | HPVs 18, 39, 58, and 59 | 1 | 0 |
|  | HPVs 31, 51, 52, and 56 | 1 | 0 |
|  | HPVs 35, 45, 51, and 59 | 1 | 0 |
|  | HPVs 35, 45, 52, and 56 | 0 | 1 |
|  | HPVs 51, 56, 58, and 66 | 1 | 1 |
|  | HPVs 52, 56, 66, and 68 | 1 | 0 |
|  | HPVs 16, 18, 51, 66 and 68 | 0 | 0 |
|  | HPVs 31, 51, 52, 59 and 66 | 0 | 0 |

^a^ Includes samples that had an HPV result by both tests and are positive for only one HPV type by Anyplex or E6/E7-NGS.

^b^ Includes samples that had an HPV result by both tests and are positive for ≥2 HPV types by Anyplex or E6/E7-NGS.

**Supplementary Table 4**: Sequence variation relative to the HPV16 and HPV18 prototype sequences, based on E6/E7-NGS detection, according to HPV test results by cobas and stratified by geographic region (Brazil/Ecuador vs. Belgium/Portugal).

|  |  |  |  | **Brazil/Ecuador** | | | | **Belgium/Portugal** | | | | |
| --- | --- | --- | --- | --- | --- | --- | --- | --- | --- | --- | --- | --- |
| **HPV E6/E7-NGS detection** | **HPV test** | **HPV test results** | **n (%)** | **Lack of homology, %^a^** | | | |  |  |  |  |  |
|  |  |  |  | **Range** | **Median** | **Q1, Q3** | **P-value**^b^ | **n (%)** | **Range** | **Median** | **Q1, Q3** | **P-value**^b^ |
| **HPV16** | **cobas**^c^  n = 144 | **+** | 55 (79.7) | 0.00 - 1.46 | 0.13 | 0.00, 0.93 | **0.0426** | 71 (94.7) | 0.00 - 1.46 | 0.13 | 0.00, 0.13 | 0.8941 |
|  |  | **-** | 14 (20.3) | 0.00 - 1.36 | 0.93 | 0.13, 1.19 |  | 4 (5.3) | 0.00 - 1.46 | 0.07 | 0.00, 0.80 |  |
|  | **Anyplex**^d^  n = 139 | **+** | 52 (76.5) | 0.00 - 1.46 | 0.13 | 0.00, 0.59 | **0.0090** | 68 (95.8) | 0.00 - 1.46 | 0.13 | 0.00, 0.13 | 0.4708 |
|  |  | **-** | 16 (23.5) | 0.00 - 1.33 | 0.93 | 0.13, 1.19 |  | 3 (4.2) | 0.00 - 1.33 | 0.00 | 0.00, 0.13 |  |
| **HPV18** | **cobas**^c^  n = 33 | **+** | 14 (87.5) | 0.00 - 1.50 | 0.37 | 0.37, 1.25 | 0.5000 | 15 (88.2) | 0.00 - 1.62 | 0.37 | 0.37, 1.25 | 0.6176 |
|  |  | **-** | 2 (12.5) | 0.25 - 0.37 | 0.31 | 0.25, 0.37 |  | 2 (11.8) | 0.50 - 0.50 | 0.50 | 0.50, 0.50 |  |
|  | **Anyplex**^d^  n = 29 | **+** | 13 (81.3) | 0.00 - 1.50 | 0.37 | 0.37, 1.50 | 0.4321 | 13 (100.0) | 0.25 - 1.62 | 0.37 | 0.37, 1.00 | - |
|  |  | **-** | 3 (18.7) | 0.25 - 0.37 | 0.37 | 0.25, 0.37 |  | 0 (0.0) | - | - | - |  |

Sequence variation was defined as the proportion of single nucleotide polymorphisms across the E6/E7 region relative to the reference sequence. HPV, human papillomavirus; Q, quartile

Significant associations are bolded.

^a^ Calculated by dividing the number of polymorphisms by the length of E6/E7 gene *100.

^b^ For the Mann-Whitney test.

^c^ Includes samples that had an HPV result by cobas and next generation sequencing.

^d^ Includes samples that had an HPV result by Anyplex and next generation sequencing.

**Supplementary Table 5**: Sequence variation relative to the prototype sequences of the 12 other high-risk HPVs, based on E6/E7-NGS detection, according to HPV test results by Anyplex and stratified by geographic region (Brazil/Ecuador vs. Belgium/Portugal)

|  |  | **Brazil/Ecuador** | | | | | **Belgium/Portugal** | | | | |
| --- | --- | --- | --- | --- | --- | --- | --- | --- | --- | --- | --- |
| **HPV E6/E7-NGS detection** | **HPV test results by Anyplex**^b^ | **n (%)** | **Sequence diversity, %^a^** | | | |  | **Sequence diversity, %^a^** | | | |
|  |  |  | **Range** | **Median** | **Q1, Q3** | **P-value**^c^ | **n (%)** | **Range** | **Median** | **Q1, Q3** | **P-value**^c^ |
| **HPV31** | **+** | 21 (56.8) | 0.00 - 1.34 | 1.07 | 0.27, 1.20 | 0.0817 | 38 (77.5) | 0.00 - 1.34 | 1.20 | 1.07, 1.20 | 0.0966 |
|  | **-** | 16 (43.2) | 0.13 - 1.27 | 1.20 | 1.07, 1.27 |  | 11 (22.5) | 0.67 - 1.20 | 1.07 | 1.07, 1.20 |  |
| **HPV33** | **+** | 4 (80.0) | 0.00 – 0.67 | 0.00 | 0.00, 0.33 | 0.8000 | 13 (76.5) | 0.00 – 1.58 | 0.53 | 0.00, 0.53 | **0.0252** |
|  | **-** | 1 (20.0) | 0.40 – 0.40 | 0.40 | 0.40, 0.40 |  | 4 (23.5) | 0.66 – 1.19 | 0.66 | 0.66, 0.92 |  |
| **HPV35** | **+** | 15 (78.9) | 0.27 - 0.67 | 0.67 | 0.67, 0.67 | 0.9391 | 10 (83.3) | 0.40 - 0.80 | 0.67 | 0.53, 0.67 | 1.0000 |
|  | **-** | 4 (21.1) | 0.67 - 0.67 | 0.67 | 0.67, 0.67 |  | 2 (16.7) | 0.67 - 0.67 | 0.67 | 0.67, 0.67 |  |
| **HPV39** | **+** | 9 (90.0) | 0.00 - 0.12 | 0.00 | 0.00, 0.00 | 1.0000 | 8 (80.0) | 0.00 - 0.12 | 0.00 | 0.00, 0.00 | 1.0000 |
|  | **-** | 1 (10.0) | 0.00 - 0.00 | 0.00 | 0.00, 0.00 |  | 2 (20.0) | 0.00 - 0.00 | 0.00 | 0.00, 0.00 |  |
| **HPV45** | **+** | 8 (88.9) | 0.00 - 0.99 | 0.87 | 0.87, 0.99 | 0.2222 | 16 (94.1) | 0.00 - 1.49 | 0.87 | 0.87, 1.06 | 1.0000 |
|  | **-** | 1 (11.1) | 0.00 - 0.00 | 0.00 | 0.00, 0.00 |  | 1 (5.9) | 0.87 - 0.87 | 0.87 | 0.87, 0.87 |  |
| **HPV51** | **+** | 7 (77.8) | 0.00 - 0.78 | 0.00 | 0.00, 0.26 | 1.0000 | 24 (70.6) | 0.00 - 0.78 | 0.00 | 0.00, 0.65 | 0.2002 |
|  | **-** | 2 (22.2) | 0.00 - 0.13 | 0.06 | 0.00, 0.13 |  | 10 (29.4) | 0.00 - 0.00 | 0.00 | 0.00, 0.00 |  |
| **HPV52** | **+** | 15 (71.4) | 0.00 - 1.20 | 0.00 | 0.00, 0.53 | 0.5887 | 21 (77.8) | 0.00 - 1.20 | 0.27 | 0.00, 0.27 | 0.7818 |
|  | **-** | 6 (28.6) | 0.00 - 0.53 | 0.00 | 0.00, 0.27 |  | 6 (22.2) | 0.13 - 0.27 | 0.20 | 0.13, 0.27 |  |
| **HPV56** | **+** | 12 (70.6) | 0.00 - 0.63 | 0.51 | 0.44, 0.63 | 0.3901 | 17 (77.3) | 0.00 - 0.76 | 0.51 | 0.51, 0.63 | 0.5944 |
|  | **-** | 5 (29.4) | 0.00 - 0.51 | 0.51 | 0.51, 0.51 |  | 5 (22.7) | 0.51 - 0.76 | 0.51 | 0.51, 0.63 |  |
| **HPV58** | **+** | 19 (90.5) | 0.26 - 1.71 | 0.53 | 0.53, 0.53 | 1.0000 | 22 (73.3) | 0.26 - 1.71 | 0.53 | 0.53, 0.66 | **0.0456** |
|  | **-** | 2 (9.5) | 0.53 - 0.53 | 0.53 | 0.53, 0.53 |  | 8 (26.7) | 0.53 - 1.18 | 0.99 | 0.59, 1.05 |  |
| **HPV59** | **+** | 21 (51.2) | 0.00 - 0.00 | 0.00 | 0.00, 0.00 | 1.0000 | 14 (93.3) | 0.00 - 0.00 | 0.00 | 0.00, 0.00 | 1.0000 |
|  | **-** | 20 (48.8) | 0.00 - 0.00 | 0.00 | 0.00, 0.00 |  | 1 (6.7) | 0.00 - 0.00 | 0.00 | 0.00, 0.00 |  |
| **HPV66** | **+** | 16 (61.5) | 0.00 - 1.40 | 0.00 | 0.00, 1.14 | **0.0238** | 19 (70.4) | 0.00 - 1.52 | 1.14 | 0.13, 1.27 | 0.2223 |
|  | **-** | 10 (38.5) | 0.00 - 1.27 | 1.14 | 1.14, 1.27 |  | 8 (29.6) | 0.76 - 1.52 | 1.21 | 1.08, 1.40 |  |
| **HPV68** | **+** | 9 (56.2) | 0.00 - 0.12 | 0.00 | 0.00, 0.00 | **0.0049** | 9 (47.4) | 0.00 - 0.12 | 0.00 | 0.00, 0.00 | 0.4211 |
|  | **-** | 7 (43.8) | 0.00 - 0.61 | 0.49 | 0.12, 0.61 |  | 10 (52.6) | 0.00 - 0.00 | 0.00 | 0.00, 0.00 |  |

Sequence variation was defined as the proportion of single nucleotide polymorphisms across the E6/E7 region relative to the reference sequence. HPV, human papillomavirus; Q, quartile

Significant associations are bolded.

^a^ Calculated by dividing the number of polymorphisms by the length of E6/E7 gene *100.

^b^ Included samples (n = 394) that had an HPV result by Anyplex and next generation sequencing.

^c^ For the Mann-Whitney test.
